# New challenges in the use of nanomedicine in cancer therapy

**DOI:** 10.1080/21655979.2021.2012907

**Published:** 2022-01-04

**Authors:** Mahmood Rasool, Arif Malik, Sulayman Waquar, Mahwish Arooj, Sara Zahid, Muhammad Asif, Sumaira Shaheen, Abrar Hussain, Hamid Ullah, Siew Hua Gan

**Affiliations:** a Center of Excellence in Genomic Medicine Research, Department of Medical Laboratory Technology, Faculty of Applied Medical Sciences, King Abdulaziz University, Jeddah, Saudi Arabia; b Institute of Molecular Biology and Biotechnology (IMBB), The University of Lahore, Lahore, Pakistan; c University College of Medicine and Dentistry (UCMD), Lahore, Pakistan; d Department of Biotechnology and ORIC, BUITEMS, Quetta, Pakistan; e Department of Biotechnology, BUITEMS, Quetta, Pakistan; f Centre for Research in Molecular Medicine (CRiMM), The University of Lahore, Lahore, Pakistan; g Department of Chemistry, BUITEMS, Quetta, Pakistan; h School of Pharmacy, Monash University Malaysia, Bandar Sunway, Malaysia

**Keywords:** Nanomedicine, active targeting, passive targeting, nanomaterials, nanoshells

## Abstract

Nanomedicines are applied as alternative treatments for anticancer agents. For the treatment of cancer, due to the small size in nanometers (nm), specific site targeting can be achieved with the use of nanomedicines, increasing their bioavailability and conferring fewer toxic side effects. Additionally, the use of minute amounts of drugs can lead to cost savings. In addition, nanotechnology is effectively applied in the preparation of such drugs as they are in nm sizes, considered one of the earliest cutoff values for the production of products utilized in nanotechnology. Early concepts described gold nanoshells as one of the successful therapies for cancer and associated diseases where the benefits of nanomedicine include effective active or passive targeting. Common medicines are degraded at a higher rate, whereas the degradation of macromolecules is time-consuming. All of the discussed properties are responsible for executing the physiological behaviors occurring at the following scale, depending on the geometry. Finally, large nanomaterials based on organic, lipid, inorganic, protein, and synthetic polymers have also been utilized to develop novel cancer cures.

## GRAPHICAL ABSTRACT

**Figure:**
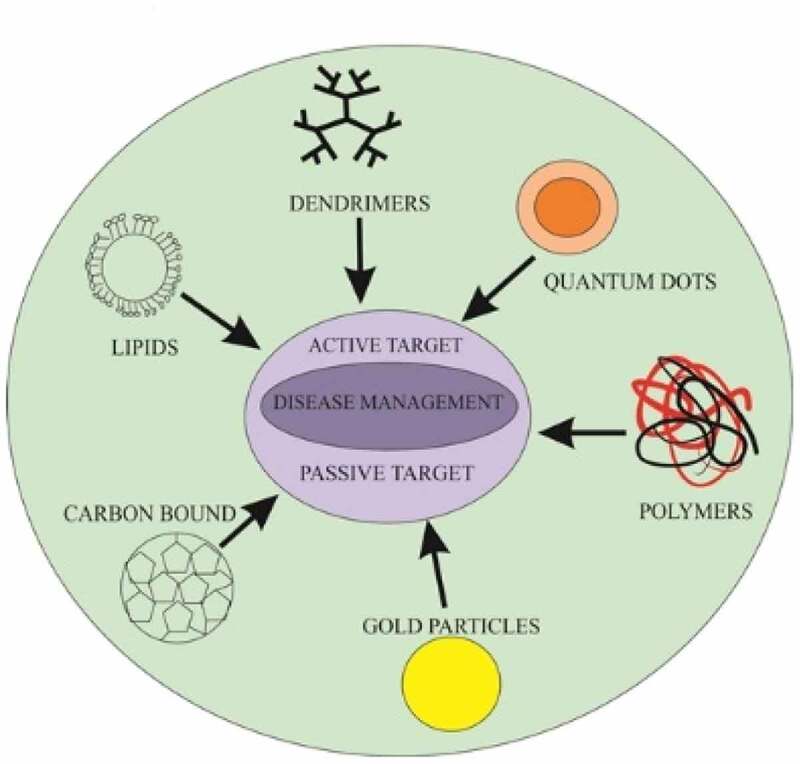

## Introduction

For the last two decades, substantial advancements have been made to understand the role of cancer biology. According to the World Health Organization (WHO), in 2012, there were 8.2 million deaths due to cancer or 13% of mortalities. However, in the following two decades, it is expected that the incidence of cancer may increase from 14 to 22 million. In addition to the challenges of developing resistance to anticancer agents, delivery of anticancer compounds to neoplastic tissue is important to limit toxicity as a result of systemic exposure. Nanotechnology plays a role in enhancing the efficient delivery of the anticancer drug to the affected tissues by increasing the efficacy and reducing the side effects [1].

Nanomaterials can benefit society in many ways, not only as nanomedicine but also in the applications of the production of solar cells or even in the efficient manufacture of more durable batteries [2]. Nanotechnology simplifies the processes involved in food and agriculture, allowing better control of their production. This approach has great potential in the application of pharmaceutical chemistry for the production of nanomedicines to treat many diseases, including cancer, which cause millions of deaths annually worldwide.

Nanomedicine involves the production of miniature-sized products with ideal properties, including reduced degradation time and decreased toxicity [3]. Several products involving the synthesis of nanoparticles or their utilization are currently in development with the higher cost playing a large role. Nevertheless, nanomedicine is becoming an attractive field for research and the pharmaceutical industry due to its higher efficacy and requirement of small quantities with better utility for drugs that are rare or expensive. Similarly, gold nanoshells are one of the most successful therapies for cancer and associated diseases. As discussed earlier, nanotechnology enhances drug bioavailability due to the increased surface area conferred by the nanoparticles. Additionally, considering nanomedicine’s effect at the cellular level, the uptake and processing of nanomedicine are dependent on several properties where physical factor size remains the most critical. Optimal uptake of colloidal gold particles was observed with one that was approximately 50 nm in size and efficiently disposed of from the body when compared to particles greater than 200 nm, as macrophages can phagocytose molecules less than 200 nm efficiently. All of the properties allow execution of physiological behaviors that remain dependent on the geometry. Many nanomaterials are based on organic, lipid, inorganic, protein, and synthetic polymers utilized in the novel treatment of cancer.

Nanomedicines can improve anticancer therapy by changing their pharmacology and improving the distribution within tissue at the site of action. The first Food and Drug Authority (FDA)-approved anticancer nanomedicine used featuring enhanced permeability and retention (EPR) was liposomal doxorubicin in 1995 [4]. Several derivatives of doxorubicin are distributed against free drugs and are approved for effective use against standard therapies [5]. Nanotechnology also overcomes multidrug-resistant cancer. Abnormal basement membranes, proliferating endothelial cells, and a lack of pericytes favor the uptake of engineered nanoparticles (passive targeting), while active targeting is based on the binding of ligands to receptors [6]. Nanoparticles are presently preferred by scientists for the reason of their high surface-area-to-volume ratio and high reactivity and are anticipated to have a better application. For example like mesoporous silica nanoparticles are used as an effective drug carrier in gastric cancer [7].

## Personalized medicine and nanotechnology: a positive venture

Mortality is significantly reduced with the use of nanotechnologies, particularly in cancer aggregation, and it can be controlled efficiently using specified diagnostic devices, specialized agents and treatment methods [8]. Ongoing procedures of cancer management include surgery, radiotherapy and chemotherapy. Among the challenges in using anticancer agents as a delivery system is the use of nanoparticles as these microsized particles are conjugated with separate ligands, as recommended by the National Institute of Health, for treating many diseases, such as cancers and cardiovascular diseases. Nanoparticles may be functionalized carbon tubes, nanofibers, or nanomachines from DNA parts and scaffolds, nanosized genetic materials and diagnostic agents [9] (Table 1). They are also categorized into actively and passively targeting particles. Passively targeted nanoparticles accumulate within the tumor and are responsible for sustainability and permeability. By 1980, these particles were considered for their clinical use and were marketed in 1990. Alternatively, actively targeted nanocarriers are conjugated with such molecules in association with antigens or receptors expressed by tumor cells. Diverse classes of nanoparticles include (1) liposomes, a lamellar structure enclosed by lipid bilayers, (2) nonionic surfactant-based vesicles called niosomes formed from hydrated surfactant monomers, (4) polymeric nanoparticles, (5) highly branched dendrimers, monodisperse, globular polymeric macromolecules, (6) lipid nanoparticles, and (7) quantum dots. The following table shows the composition, structural architecture, types and application of the various nanoparticle classes (Zazo et al., 2016; Didem et al., 2016; Selvarajan et al., 2020; Akbarzadeh et al., 2012).

**Table 1.** List of nanoparticle types, their composition and properties with respect to drugs

Nanoparticle type	Composition	Properties/applications	Types and Structural architecture
**Organic polymers**	Composed of Phospholipids and Cholesterol. Bilayered lipids containing enteral aqueous portion. The lipids are cationic and neutral of phosphatidylcholine class and sterols	Biodegradable, biocompatible, nonimmunogenic, amphiphilic, alternating lipid modification and drug delivery capacity	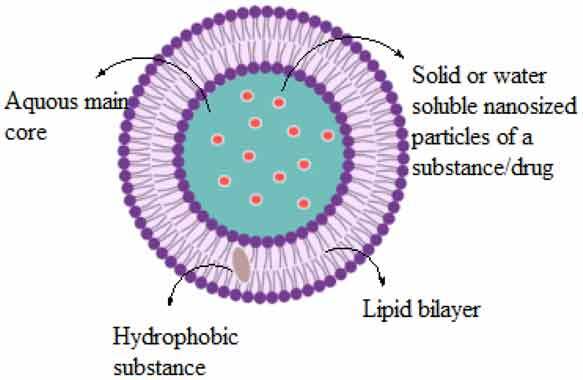
**Liposome**			
**Niosomes**	Formed by self-association of nonionic surfactants and cholesterol in an aqueous phase.	Biodegradable, biocompatible, have nonimmunogenic skeleton, and therefore serve as promising drug carriers	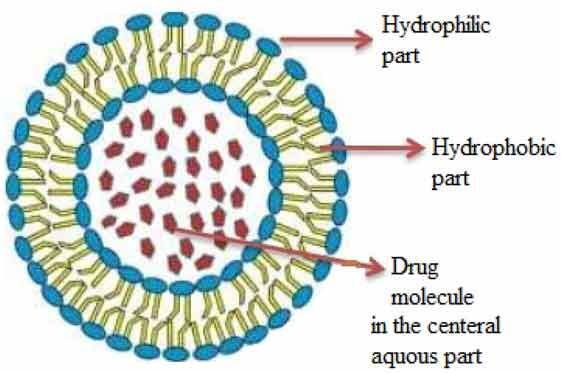
**Polymeric nanoparticle**	Polymers with suspended nanosized substances/drugs	Synthetic and natural origin biodegradable and biocompatible polymers are used	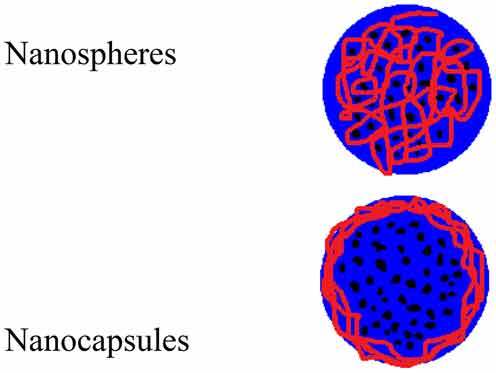 Nanospheres Nanocapsules
**Dendrimer nanoparticles**	Polymeric monomeric or oligomeric core shell nanostructure having a substance/drug encapsulated in their branches or absorbed on their surface	Biocompatible, has low polydispersity, and can be hydrophobic as well as hydrophilic	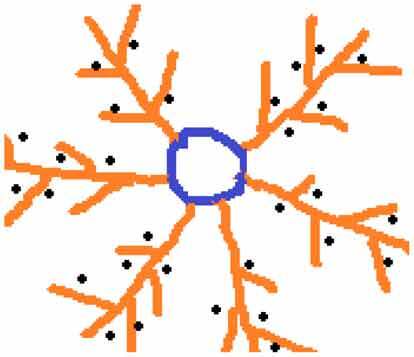 Conjugated type and Encapsulate type
**Lipid nanoparticles**	Lipids combined with drugs or spherical vesicle nanoparticles made of ionizable lipids, positively charged at low pH (enabling RNA complexation) and neutral at physiological pH	Attractive drug carriers due to easy manufacturing, scale up capacity, biocompatibility, and good biodegradability	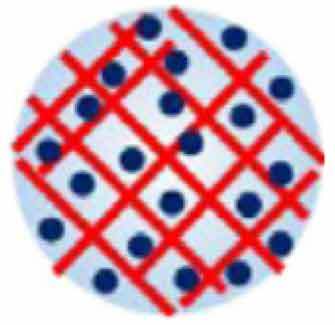 Solid lipid Nanoparticle (SLN), nanostructured lipid carriers (NLCs) 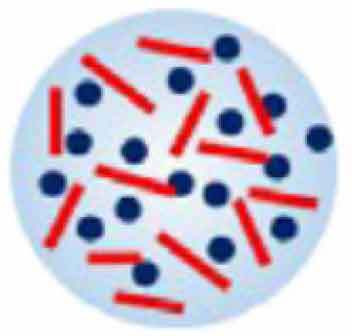 and lipid drug conjugate
**Carbon based nanoparticles**	Pure carbon	Highly electrical, heat conductor, high stability, low toxicity, biocompatible, application in drug delivery	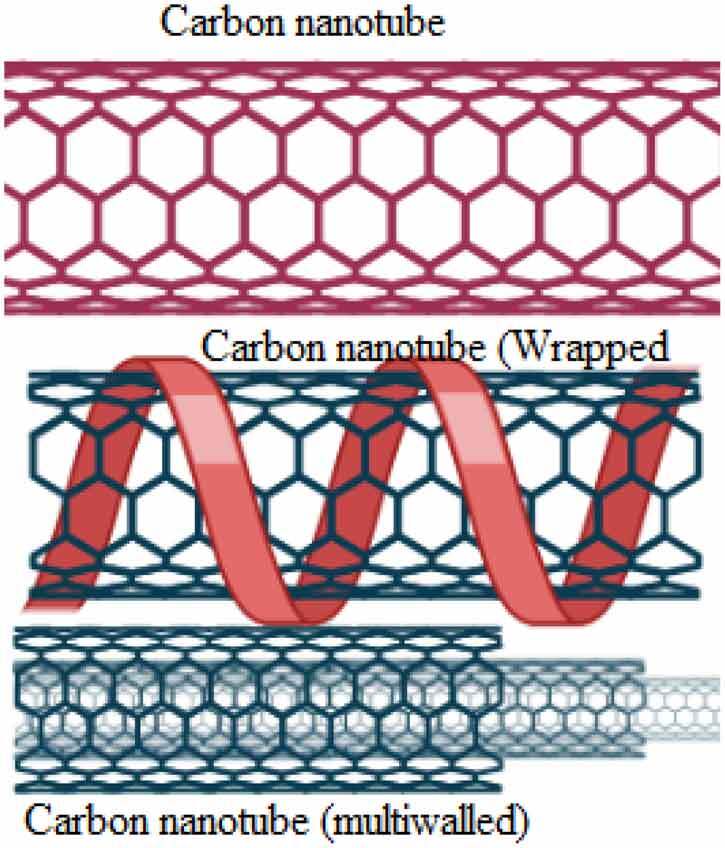
**Inorganic nanoparticles**: These include quantum dots, polystyrene, magnetic, ceramic and metallic nanoparticles, and have a central core composed of inorganic moieties that are responsible for their fluorescent, magnetic, electronic and optical properties.			
**Quantum dot nanoparticles**	Semiconducting nanoparticles, artificial nanostructure metalloid crystalline core usually manufactured from CdSe or CdTe surrounded by a zinc sulfide (ZnS) outer shell	Electron transporter, they can emit light of various colors. They have applications in composites, solar cells and fluorescent biological labels, optical imaging	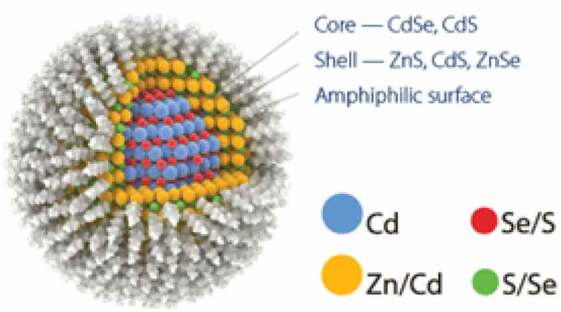
**Gold particles**	Gold combined with a substance/drug	Biocompatible, within the range of 5 to 100 nm, in the drug delivery system	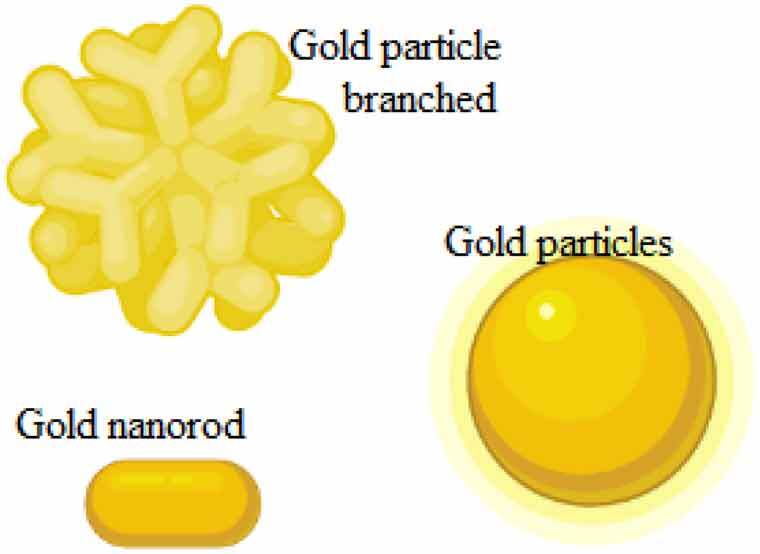
**Silica nanoparticles**	Composed of an amorphous network of silicon and oxygen	Biocompatible, large surface area, easy functionalization, bioactive and commonly used in drug delivery and contrast imaging	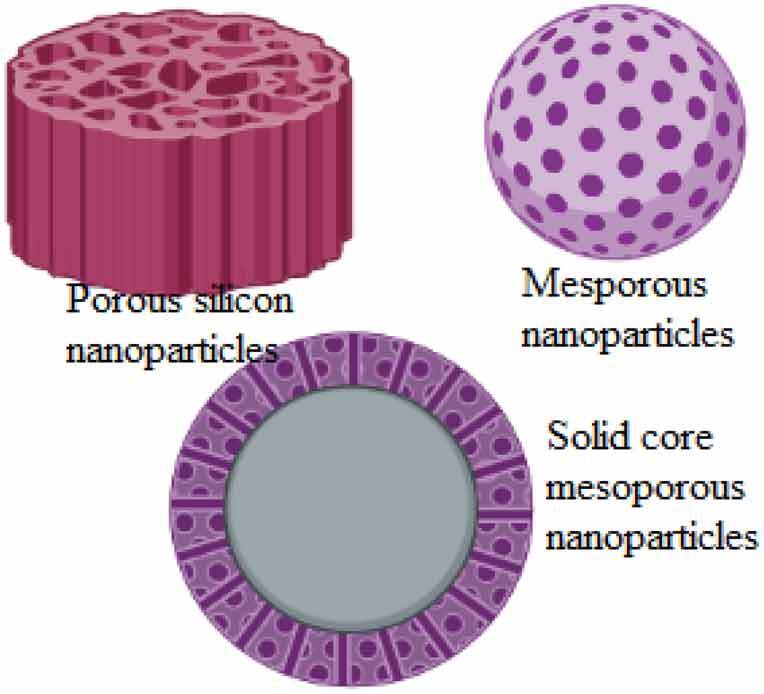
**Magnetic nanoparticles**	They contain major components, such as magnetite, Fe, Ni, and cobalt	Ferromagnetic, contrasting agent in the case of MRI and certain therapy	Cobalt based, Sphere 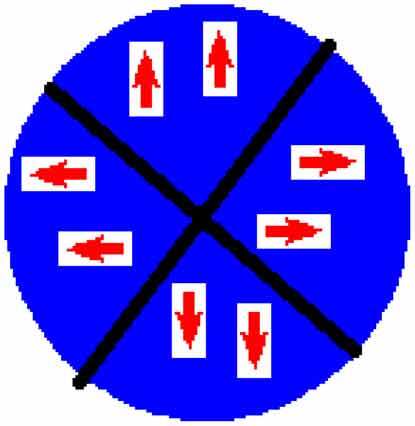

## Passive targeting

Most nanocarrier-based cancer cures are passively targeted first-generation nanomedicines. First generation nanomedicine drugs rely primarily on manipulating the pharmacokinetics and biodistribution by regulating physicochemical properties [10]. Examples of first-generation drugs based on inactive targeting are pegylated liposomal doxorubicin and nab-paclitaxel. Cancers’ pathophysiological features and their surroundings have been used for inactive targeting, particularly where the accumulation of nanomedicine in cancer cells is further promoted by the EPR effect. Therefore, through diffusion and convection, nanomedicine treatments from passive targeting into neoplasms can occur without the attachment of a particular substance to the nanocarrier surface. Nevertheless, it has been widely accepted that EPR effect-based passive targeting is insufficient to control cytotoxic drug side effects, and there are greater benefits using directed delivery.

Cancer heterogeneity and its stroma, such as hypoxic slopes, can negatively affect the delivery of drugs through passive directing, resulting in reduced or abolished transport of compounds into neoplasms [11]. Recently, researchers have focused on the standardization of neoplasm vasculature before starting cancer treatment. Moreover, drug penetration is limited to the tumor due to extracellular matrix-like pancreatic cancer [12]. Additionally, the accumulation of nanocarriers is not prevented by passive targeting in former organs of the fenestrated endothelium, such as the spleen and liver [13], justifying the next-generation development of nanomedicine with advanced practicalities. The basis of nanomedicine second-generation technology is drug delivery with active directing transmitters or nanocarriers having stimuli-reactive properties. Therefore, improved directing and enhanced efficiency potential is maintained by second-generation nanomedicine [14].

## Active targeting

In active targeting, a high-affinity substance attaches to the nanocarrier surface. The ligand selectively binds to the target cell receptor [15]. A wide ligand range, such as carbohydrates and folic acid, or macromolecules, such as amides, proteins, oligonucleotides, and aptamers, has been utilized for this purpose, which includes small particles of substances. The preferred ligand binds to a targeted cell while minimizing binding to healthy cells.

## Using animal models for testing nanomedicines

Due to the relevance of the primary stage of clinical and preclinical data for nanomedicines, we were not successful in determining the efficacy and toxicology of the employed nanomedicines. One of the essential determinants of the distribution of nanoparticles in the mononuclear phagocytic system (MPS) remains associated with MPS organs in common laboratory animals and is believed to be Kupffer cells of the liver and spleen, similar to humans. Another observation is the use of preclinical species, for example, goats and pigs, where the distribution of particles into pulmonary intravascular macrophages is also observed. Intravenous injection of certain nanosized particles (such as iron oxide particles) leads to a higher uptake in the pulmonary intravasculature (less than 85% of dose) in sheep, calf, pig and goat models. Consequently, the second greatest uptake was observed in the Kupffer cells of the liver and spleen, which may be less than 65% of the dose, in animals such as monkeys, hyrax, rabbits, and Guinea pigs [16].

The application of conventional species for toxicological and pharmacokinetic evaluation of certain nanomedicines remains the similar type of MPS profile. Based on certain statistical techniques, such as allometric analysis, the clearance of pegylated TNF (gold particles) executes a similar response. However, apart from the species, these responses remain dependent upon the physical properties of the respective subject; for example, clearance brain weight products remained proportional to the body weight of selected species. Additionally, they proposed common mechanisms of nanomedicine disposition. Several recent studies state the clearance of pegylated liposomal anticancer agents [17]. The accumulation of these nanoparticles in organs, especially MPS-targeted tissues, is common in the case of nanomedicine due to repeat-dose distribution of the tissues and the presence of metals and nonbiodegradable polymers. General evaluation describes the distribution of tumors and the efficiency of oncodrugs. Another unique tumor distribution of small molecule nanomedicines is dependent on long systemic circulation and vascular permeability for their uptake in interstitial spaces. Studies have explained the longer circulation of pegylated nanoparticles, which have an increased concentration and exposure confirmed by AUC (area under the concentration curve) [18]. However, there are no systemic studies evaluating the clinical relevance of vascular permeability in animal models. The small pore sizes of nanomedicine allow tumors to vesiculate. Moreover, its histological types are dependent on tumor implantation, as in brain tumors, due to very low permeability compared to peripherally implanted tumors, suggesting the significance of the tumor microenvironment in influencing vascular permeability. Primary studies have suggested that these tumors have some ultrastructural features that may be defined as fenestrations, as seen in other animal models. However, other cancer types in humans such as brain tumors are mostly devoid of any pores, and the cancer vascular permeability differs significantly from those in animal models. Specific animal models should be chosen to evaluate the activity of a certain type of cancer regardless of nanoparticle permeability. A similar recommendation is made for nanosized molecules that are efficiently used in different models and are therefore applied in nanomedicines [19].

Previous studies have revealed the novelty and application of small molecules against various diseases; however, major issues related to cancer nanomedicine and its development are immunological and hematological complications. Certain types of reactions known as anaphylactic reactions are of initial concern for the translation of iron oxide nanoparticles. These concerns are related to the polymer coatings, which are used to remove agents found in their commercial forms. Additionally, endotoxin contamination and associated complication activation and pyrexia are vital issues in using nanomedicine for treating cancers and as a therapeutic agent. Therefore, limitations of using certain species, such as rabbits, exist in addition to the models used previously. A meta-analysis comparing the issues relating to small molecule anticancer agents in preclinical models of phase I showed that the number of dose levels and time required to complete were increased for nanoparticles compared to small molecule drugs [20].

## Preparatory methods of nanomedicines

Data indicate that if the standard animal model is obtained to define the dose in phase I of the clinical study, then small molecules are optimal nanoparticle agents. The use of intraperitoneal drugs in place of intravenous drugs may have slight consequences and may be useful for evaluating drug efficacy when applied in small animal models, whereas their higher permeability through the tissue remains a significant feature of many nanomedicines. The size of the nanomedicines is most critical in designing a therapy for cancer as a substantial difference exists between intraperitoneal and intravenous routes of administration. Therefore, it is necessary to include both nanoparticle and nonnnanoparticle formulations of drugs as controls to determine the related toxicities. Nanosized particles are unique, as discussed and explained by the number of evaluated studies and models, i.e., the production of nanoparticles requires a set of specialized methods, of which one of the most common methods remains self-assembly to amphiphilic lipids, polymers or drug conjugates [17]. A unique and novel method of nanoprecipitation can be oil in water, also termed single emulsion and double emulsification, and more precisely may be water in oil in water, called the W/O/W method [21].

The development of the most recent nanoparticles also includes this method, which is being followed by microfluids and can manipulate volumes on a nanoscale. These approaches also offer effective control and manipulation of fluids to create nanoparticles. Such fluids also offer advantages such as a large surface area and surface-to-volume ratio, which is an important factor in promoting the yield of nanoparticles of a certain uniform size. Other advantages of such fluids include their reproducibility and rapid fabrication, and they employ the application of 3D hydrodynamic focusing, which is responsible for the production of nanoparticles of several different sizes. These microfluids provide significant and rapid means for encapsulating drugs, which is not feasible through conventional approaches. To obtain the maximum advantage of such formulations, one must tackle the limitations of higher cost and large-scale production, making it feasible for use as a clinical agent [20].

Drugs synthesized by nanotechnology protocols have the potential to be modified using certain standards, such as PEGylation. Currently, gold nanoparticles are being modified by polyethylene glycol (PEG). PEG serves as a functional agent in reducing protein absorption, particularly apolipoprotein and complementary protein C3, through hydrophobicity and steric repulsion effects, which tend to extend the circulation time in blood, allowing nanoparticles to persist in the bloodstream for an extended time; therefore, these nanoparticles are recognized at the therapeutic site of action. Nanoparticles are resistant to protein adsorption by electrostatistically-induced hydration. This moiety allows alteration and recognition of the nanoparticle surface by tumor cells based on pH differences between normal cells and the microenvironment of the tumor surface charge, allowing for efficient cellular uptake compared to highly hydrophilic PEG nanoparticles. The highest circulation time that can be obtained from synthetic particles is under 300 hours in clinical trials, while human red blood cells circulate in the body for approximately 100–120 days. This fact is mainly dependent upon the membrane proteins CD47, which are also one of the self-markers on the cell membrane. Studies have revealed that nanoparticles have a longer half-life than PEG-coated nanoparticles (NPs) [22].

Nanoformulations have become an important tool to review promising entities with reduced efficacy due to poor pharmaceutical properties, such as cytotoxicity or poor uptake of cellular products. One example is CRLX101, which is a polymer-based nanoparticle composed of the conjugate of camptothecin (CPT) to a cyclodextrin-containing polymer (CDP) that acts against solid tumor aggregation. However, some toxic effects led to changing the development of CPT to attain efficacy in tumor suppression. CPT have a sustained profile after being released in the intracellular spaces, associated with decreased toxicity, and decreased levels may be observed in phase I/II studies. Many promising drugs, such as camptothecin and wortmannin, do not achieve the desired results and are not stable or soluble, whereas nanomedicines are still believed to have the potential to overcome these problems, especially in the case of anticancer drugs. Nanomedicine is used worldwide due to it having less lethal and toxic results compared to other conventional cancer therapeutics that have more adverse effects [23].

## Advanced personalized nanomedicine for disease treatment

Epigenetic changes that can be characterized by methylation and modification depend on the heritable design of different genes. While there remains no impact on the sequences of genes, such changes can specify the patterns of growth and differentiation. These genetic variations are responsible for the fertilization of a single egg upon exposure to genetic material, and likewise, the development of a complicated biological system is initiated, such as the organization of several different tissues. Different organs are produced, and then a complete biological system is formed. The primary role of genetics could be seen in the physiology and pathogenicity of the pathogen; for example, there is the chance of large variations between monozygotic (MZ) twins if they are allowed to grow separately, while the same behavior could be attenuated if they are grown in similar conditions [24]. Although the genetic makeup of all individuals is believed to be 99.9% similar, the novelty of 0.1% can differentiate one person from another.

Disease development is dependent on genetics and other pathophysiological causes, and if someone encounters an imbalance in physiology, which could be either through a genetic cause or other reasons, cancerous lesions may develop. Conventional techniques to tackle such conditions rely on principle or well-known fact as every disease has only one appropriate target for its proper handling, whereas selection of treatment type remains dependent on the one’s physiology and previous medical records. Although such approaches can treat cancers (i.e., testicular cancer and leukemia) [25], they were not enough to cope with the increasing rates of cancer. Therefore, developing a more significant and proper approach to manage infectious diseases is urgently needed. New approaches should have a comprehensive knowledge of biology, ongoing therapies and appropriate delivery mechanisms and should be aware of the individuals’ age, sex and physiological conditions, leading to the development of personalized medicines that will be more specific to the individual.

Such approaches would especially emphasize the uniqueness of a person, disease, and drug with reduced side effects and comprehensively participate in managing numerous lethal diseases, particularly cancer [26]. An ideal example for the management of diseases is the approach in which ER (estrogen receptor) is identified by appropriate protocols within patients with breast cancer. Among the list of several biomarkers, vital biomarkers have been found to help clinicians determine the appropriate drug delivery method to attain maximum efficacy. As discussed, these vital markers are epigenetic modifiers but are associated with limitations of inappropriate delivery systems due to their higher molecular mass and structures. Hence, to overcome these issues, there is a need to design microsized medicines with efficient and instant delivery systems. Newly synthesized nanomedicines would be easier to deliver with the help of specific nanoimaging reagents and specified devices. The application of a nanomedicine is not limited to the management and control of a few diseases but can be used against various diseases, among which cancer therapies are of prime concern [27,28].

## Principle for nanomedicine product development for cancer therapy

There are many statements in favor of nanosized therapeutic development [27]. First, nanoparticles might overcome solubility and stability problems of anticancer drugs. The compound’s bioavailability is limited by water solubility, and the development of early agents of anticancer drugs might be hampered. Encapsulating the compound inside a hydrophilic nanocarrier increases the delivery and consumption of poorly soluble drugs. Simultaneously, chemical stability increases. One of the examples of drugs having inadequate stability and solubility is the P13 K inhibitor and radiosensitizer wortmannin, of which the development has ceased. The wortmannin solubility was enhanced from 4 mg/L to 20 g/L through the system of lipid-based nanocarriers in vivo, while stability was also increased [27].

Second, nanocarriers can defend anticancer compounds from excretion or decomposition, and therefore, the pharmacokinetic visibility of compounds can be determined, as enzymatically cleaved drugs (for example, within plasma siRNA through RNAses, within stomach proteins through trypsin or pepsin) can be kept from being degraded through enzymes. This problem can be resolved through encapsulation of antineoplastic agents in nanocarriers or matching perishable compounds to synthetic compounds. Third, the distribution and direction of anticancer medicines can be improved through nanotechnology. Antitumor drug distribution is set through their physicochemical properties, and drug insight is limited to neoplasm tissue. [29]. Construction of nanomedicine can help to ameliorate drug penetration and redirection or selectively direct compounds to cancer cells or stromal compartment cells. For redirection of antitumor drugs, both active and passive directing schemes are used.

A fourth reason in favor of nanomedicine-based therapy development is that nanocarriers are designed to expel their payload on initiation and thus result in stimuli-sensitive nanomedicine treatments; for example, drugs independent of pH, such as doxorubicin, can be coupled on pH-sensitive nanoparticles to increase the cellular uptake and intracellular release of drugs [30]. Ultimately, the resistance of neoplasms is reduced against antitumor drugs through directed nanomedicine treatments. In general, nonspecificity was reduced by targeted intake and MDR/ATP outflow pump-driven excretion. Thus, the circulation time of a compound can be sustained by nanomedicine, helping the release of stimuli-responsive drugs to mediate endocytic intake of the drug. In any cancer therapy, a balance is must be maintained between the potential harms and benefits of treatment. The objective of nanoapplications is shifting this balance toward benefits. The following are the general schemes and advanced practicalities of nanomedicine products to ameliorate the therapeutic index of the drugs.

## The use of personalized medicine for cancer

Even though every person has a very similar genetic makeup, everyone remains unique. Therefore, the use of personalized medicine such as a drug that remains specific to each patient and its physiology are important. Such nanobased drugs have displayed several positive results. The identity of each patient is refers to their specific environment, surroundings and unique physiology regarding how they will react to certain therapies or drugs. Patients are also subjected numerous onco-drugs and their responses are studied. Personalized medicines used against cancer and other diseases have been recognized for their maximum therapeutic index and for curing toxic diseases [31]. Administering medicine to an individual specifically based on his or her sex, nutrition and race should lead to the optimum results and maximum efficacy. In the case of cancer therapy, such medication is termed personalized oncology. Cancer is considered a disease that surpasses cardiovascular disease. Such therapies have a broader spectrum, as they mainly focus on genomic and epigenomic abnormalities and show dual effects, i.e., reducing toxicity and providing better and more efficient results [32].

### 1) Role of nanoparticles in the delivery of drugs

In the conventional treatments of cancer, a patient who undergoes surgery for a specific tumor (especially if the tumor is malignant) is prescribed specific postoperative protocols. For chemotherapy, a particular toxic chemical is introduced into the patient’s body at regular intervals so that the remainder of the proliferative cells within the body are targeted. However, this technique has disadvantages, such as its lethal effect on normal cells within the body. Accordingly, therapy provided for the tumor cells is lethal for the normal cells, leading to neural toxicity, suppression of bone marrow and cardiomyopathy, etc. Such adverse effects of chemotherapy could be efficiently decreased by using nanotechnology, because we could effectively design particular complex nanoparticles that specifically target the tumor site and hence show no or comparatively negligible adverse effects to the surroundings [33].

### 2) Application of nanoparticles in identifying biomarkers

Recently, several nanotherapies have been developed, and such therapeutics have been applied to a group of patients suffering from cancer, but only a few approved carriers used specifically target cancer cells [34]. There are individual markers that serve as a key for personalized therapies, as they can distinguish between tumors within patients at a particular stage, making it easier to predict possible outcomes. Nanoparticulate systems are a valuable tool for identifying diagnostic and prognostic markers, for detecting tumor cells within the body, and for testing the efficiency of particular therapies in the case of different diseases. Such systems may include the use of quantum dots, biocompositing, etc. These techniques have great potential in detecting the initial causes of numerous tumors [35]. Another milestone beyond the discovery of sensitive diagnostic tools uses nanobiomarkers in detecting various clinical samples and can also capture such cells in the bloodstream [36].

Hence, using nanobiomarkers to detect tumor growths, particularly when they is not detectable using other typical techniques is notable. To date, nanotechnology has several benefits in formulating personalized treatment. This technology is an efficient way to deliver drugs to a targeted site. Certain miniature-sized devices, such as nanochips and bionanosensors, are installed in the body to improve drug delivery at the targeted site and could prove to be an efficient prognostic and diagnostic tool [37]. Although there are several imaging techniques used to detect tumors, advancements in the field of nanotechnology have resulted in novel molecular imaging tools (e.g., perfluorocarbon nanoparticles) that can detect even extremely small tumors in their prestages. However, there are several limiting factors associated with the use of this technology on a larger scale. One limitation is the lack of efficient ways in determining the pattern of how a nanoparticulate will distribute in the body once they are administered [38]. Moreover, some of the nanoparticles are highly toxic to some organs, and some are found to have less solubility or no biodegradation, which limits the use of these particles on a larger scale. While there are associated limitations of nanoparticles, there are still some FDA-approved particles that can be used with less adverse effects, including liposome-encapsulated doxorubicin. This nanomedicine revolutionized the use of nanosized particles in managing several lethal diseases, such as cancers, with less toxicity (e.g., cardiotoxicity), though it was not highly efficient against tumors [39]. Following the new trends for designing medicines against infectious diseases, trials have continued over the last few years to study the use of small molecules for therapeutic purposes. Every year, thousands of such remedies are introduced and go through clinical trials, and few receive approval and are used significantly. Nanotechnology has proposed new modes in cancer therapies to provide successful treatments and improvements [40]. Some of those improvements include A) the delivery systems of drugs, B) minimization of the emerging toxic effects of the medicine and finally, overcoming the problems of low drug bioavailability.

Interest in lipid-based nanomedicines, including liposomes, lipid-core, solid lipid nanoparticles, etc., has significantly increased due to their higher biological compatibility, efficient control over their properties and, most importantly, large-scale production due to their cost efficiency [41]. All types of nanomedicine have targets and are essentially dependent on passive targeting, also known as the EPR effect. Regarding tumor vasculature, abnormal fenestrations can be observed through circulating nanoparticles. Regarding active targeting, these nanomedicines can provide an essential and additional mechanism responsible for targeting nanoparticles that are mediated by binding to specific composites such as α_v_β3 integrins and folic acids [42].

Several ligands, including numerous antibodies, peptides, aptamers and some small molecules, have considerable targeting potential, but such targeting also requires an essential balance between ongoing systems, such as a proper equilibrium between ligand contents and surface exposure. Such ligands and their balance are responsible for minimizing immunological recognition, clearing nanoparticles and providing a vivid image relating to the circulating time of the particles to the targeted site. Scientists are still trying to identify more reasonable nanoparticles to treat certain diseases, such as cancers and diseases of the lungs, breast, colorectal, prostate, etc. Although there are some vaccines currently available for managing such contagious diseases, these methods are insufficient in delivering the required quantity of drug to the target site, resulting in toxic metabolites. Hence, to minimize such adverse effects, nanomedicines are designed to improve the therapeutic properties of drugs with the least toxicity to normal tissues [17, 43].

An interesting fact remains that if effective, one could increase the amount of drug delivery to its target site; for example, if only 3% of the amount of drug delivery increases, it would result in a dramatic increase in the efficacy of the treatment. Due to the unique pharmacokinetic characteristics, the action of nanomedicines remains completely different from other vaccine and drug systems. These characteristics are purely dependent on the physiochemical nature of a nanoparticle, including its size, nature, shape, density, stability and, most importantly, its route of delivery. The route of delivery plays an essential role in the delivery of drugs. A drug administered through the intramuscular route or the intravenous route has considerably different rates of absorption or bioavailable amounts. Therefore, a drug administered through the intramuscular route can be bioavailable in amounts of 1%, while it can be increased to 100% just by changing the route to intravenous. Primarily, nanoparticles accumulate within the liver, spleen, or tumor site of the patient, whereas the accumulation of nonnanoparticles is unknown [44]. In addition, the development of PEG nanoparticles beyond treating cancer patients is dependent upon the fact that the PEG form of medicine has a prolonged exposure to plasma and an enhanced amount of drug delivery compared with the amount of drug delivered in the non-PEGylated form [41].

Many formulations can consist of different drugs. The efficacy of the therapy may be increased by using several different encapsulation/conjugation combinations where the difference may be dependent upon the different hydrophobicity/hydrophilicity of the administered drugs [45]. Variability exists in the pharmacokinetic disposition of nanoparticles, but it increases greatly in the case of nanoparticles compared to nonnanoparticles. Hence, there is a need to identify the factors beyond the discussed variability, and from numerous studies, the factor may be the liposomal agents, and other associated variables, such as age, sex, body composition, and presence of a tumor within the liver.

When patients were divided into two groups, one with an age less than and equal to sixty years and the other with an age greater than sixty years, the amount of cleared PEGylated liposomal agent was 2–3-fold less in patients aged less than or equal to sixty than in patients aged above sixty. Another study regarding body composition as a variable reported on patents with lean structure and nonlean bodies. The obtained results revealed that lean structured patients presented more plasma exposure than other groups. In addition, a gender-based study concluded that the clearance rate of encapsulated PEGylated drugs was lower in women than in men. Finally, patients with tumors in their liver had lower clearance than those without tumors, because patients with tumors induce MPS, leading to an increase in their clearance [44–46]. Studies that could explain the mechanism of drug clearance and identify several factors that are specific to their pharmacokinetic and pharmacodynamic variability [44] are still needed.

The success of therapies used in for cancer patients requires significant efforts, including the development of simple, scalable, and inexpensive protocols to synthesize medicines and control biological behaviors. Nanomedicines, which are generally used as one of the most essential parts of the treatment, must be used according to the guidelines of regulatory authorities for industrial cultures prepared for the synthesis of nanomedicines, including suitable testing and characterization, to cope with upcoming challenges. The most common targets of nanomedicines include several tumors, such as breast and prostate cancers [45]. Some issues specific to nanomedicine for tumors are its distribution compared to small molecules, which are subsequently dependent on the circulation, and its permeability in vessels for uptake into the interstitial space. Numerous studies have revealed an association between PEGylation and small molecules. Nanomedicines allow for permeability into certain tumors that remain directly proportional to the size of the pore. Another essential model revealed a dependence on orthotopic brain tumors. Other preliminary studies have shown a dependence on clinical tumors and certain features, such as fenestrations, or other tumors, such as brain tumors [47]. Human cancer compared to animal models has not been thoroughly evaluated. Published reviews can explain the common regulatory framework for determining the safety related to these small molecules, related biologics, and employed devices that are sufficient to explain the use of nanomedicine.

## Role of nanotechnology in the laboratory

Nanotechnology is considered one of the alliances for cancer nanotechnology and is the source of preclinical development of nanomaterial-based drug delivery and agents. Moreover, clinical assessments are important in standard protocols and reference materials, which play a significant role in educational efforts that enhance the field of nanomedicine. A critical element beyond the successful commercialization and synthesis of such pharmaceutical products is a process that can be repeated. Additional challenges under the process of their production include sterility (the most essential parameter), nanoparticle size, encapsulation ability, removal of free drug and drug release rate. Acquiring sterility is challenging because heat or other parameters can affect size and polydiversity. If the particle size is greater than 100 nm, the polydiversity is broader where sterile filtration is not possible, leaving only a few options available, including sterilization of raw materials and aseptic processing, which are costly, and many nanoparticles fail to withstand. Isolation of unencapsulated drug from nanoparticle drug products is more difficult, and therefore, unencapsulated drug contamination compromises both safety and efficacy [45].

Similarly, if the amount of synthesized drug cannot be limited from the nanoparticles, then its performance will remain unreliable and is potentially unsafe, as it may burst and release more drug from the carrier. Actively targeted nanomedicines require a well-controlled process that provides consistent exposure to a ligand or other particles on their surface [48]. Nevertheless, different authorities, such as the FDA, have developed different scales to characterize specific drugs and the biocompatibility of different materials, purity and sterility, which may include size, surface ligand density, surface charge, and area [49]. Additionally, there is a need to measure the stability of a formulation’s time, temperature, pH, light, diluent, lyophilization, and centrifugation under suitable methods that can predict biological effects as multifunctional nanomaterials are intended to deliver drugs but face challenges that may affect its synthesis and purification.

Cancer is one of the main causes of mortality worldwide, affecting over 10 million patients every year. Currently, the treatment options include surgical resection, radiation, and chemotherapy. Although over 90 chemotherapeutic drugs have been clinically approved by the FDA, their efficacy has been severely hindered by dose-limiting toxicity and patient morbidity. Recently, there has been a new trend of using nanomedicines through nanosystems as therapeutic agents. Compared to conventional small molecule-based therapy, nanoparticles have several potential advantages for cancer therapies, including higher payload capacity, prolonged blood circulation, low toxicity, and improved antitumor efficacy.

The use of polymeric cells for cancer treatment was first reported in the early 1980s. These particles are nanosized ranging from 10–1100 nm and are also termed supramolecular constructs formed by the assembly of biocompatible amphiphilic block copolymers in aqueous environments. Currently, the most commonly used corona-forming polymer is polyethylene glycol (PEG), with a molecular weight range from 2–15 kDa. Core-forming blocks typically consist of poly(propylene oxide) (PPO), poly(D,L-lactic acid) (PDLLA), poly(ε-caprolactone) (PCL), and poly(L-aspartic acid) [33].

Paclitaxel is a highly effective anticancer agent that inhibits molecular growth by binding to the beta subunit of tubulin with a water solubility of 0.0015 mg/ml. The degree of water repellency is favorable for drug permeation through the cell membrane intravenously. The degree of solubility is enhanced and results in rapid drug aggregation and capillary formation. Through drug encapsulation within a hydrophobic core, the apparent solubility of the drug is significantly increased. Hence, polymer micelles allow for in vivo use of current drugs otherwise deemed too hydrophobic or toxic without having to manipulate the chemical structure. Additionally, encapsulating the drug within the polymer core affords drug stability by hindering enzymatic degradation and inactivation [50].

Polymer-drug conjugates, dendrimers, and liposomes represent other major polymer-based nontherapeutic systems with different chemical structures and biological properties. Among these systems, systemic liposomes have a longer history of development and are the most successful in clinics. For example, SMANCS, a conjugate of neocarzinostatin (NCS) and poly(styrene-co-maleic acid) (SMA), was developed by Maeda et al. in the 1980s. In blood, SMANCS has a 10 times higher half-life than NCS. An important effect is to improve their solubility, and tumor selectivity leads to excessive tumor formation during hepatocellular carcinoma [51]. Other types of polymer drug conjugates include dextran-doxorubicin, PEG-camptothecin, and polyglutamate–paclitaxel conjugates, which are in phase I, II, and III clinical trials. In clinical trials of doxorubicin-containing PEGylated formulations, Doxil was used to treat Kaposi’s carcinoma and several other severe types of tumors. While dendrimers have not been clinically used, preliminary research with methotrexate-containing polyamidoamine dendrimers has shown low growth of subcutaneous tumors [52].

Liposomes are vesicular nanostructures self-assembled from phospholipids and cholesterol molecules, typically from the cell plasma membrane. Due to the inner hydrophilic compartment, liposomes are more efficient in their solubility within the hydrophobic bilayer membrane, such as therapeutic proteins or DNA. Poorly soluble drugs are trapped within the hydrophobic bilayer membrane, with limited loading capacity due to membrane destabilization effects. Polymeric cells provide a unique and complementary nanoplatform for nanosystems for drug delivery applications. The hydrophobic cores provide a natural carrier atmosphere, allowing easy encapsulation of poorly soluble anticancer drugs. The noncovalent encapsulation strategy makes it feasible to trap drugs without requiring reactive chemical groups. There is unique chemistry present between the polymer’s constituents, allowing chemical conjugation in the anticancer drug system. One of the important medicines used is doxorubicin. This approach can enhance the drug development system and premature release of drugs upon administration [53]. Several other biomolecules, such as carbohydrates, galactose and lactose, have also been used in functionalized micelles. These biomolecules act as ligands and have a high affinity for the asialoglycoprotein receptor (ASGPR) overexpressed in hepatocellular carcinoma. A galactose-labeled poly(ethylene glycol)-co-poly(γ-benzyl L-glutamate) block copolymer was used by Cho et al. to produce micelles encapsulating paclitaxel [54].

## Conclusion

Nanomedicines have considerable potential for numerous infectious diseases. Efficient medicinal value can be achieved through nanomedicine due to its small size and high bioavailability at the site of action. In addition, they can reduce the toxic effect of the drug and are considered economical, particularly when a high amount of a certain drug is required as nanomedicine requires a very small amount. Hence, nanomedicine is considered one of the vital therapeutic techniques for several diseases, especially for cancer, and can reduce the cost of treatment. This significance suggests that nanomedicines are an emerging field that will be an alternative to conventional therapies/therapeutic agents in targeting various diseases efficiently, particularly cancer. Nanomedicine development will revolutionize the healthcare sector to overcome major health issues.
